# Nomograms incorporating hsa_circ_0029325 highly expressed in exosomes of hepatocellular carcinoma predict the postoperative outcomes

**DOI:** 10.1007/s12672-024-01060-7

**Published:** 2024-06-05

**Authors:** Kun-Li Yin, Taiwei Sun, Yu-Xin Duan, Wen-Tao Ye, Ming Li, Rui Liao

**Affiliations:** 1 Department of Hepatobiliary Surgery, The First Affiliated Hospital of Chongqing Medical University, No. 1 Youyi Rd, Chongqing, 400016 China; 2 Department of Radiation Oncology, Zhongshan Hospital, Fudan University, Shanghai, China

**Keywords:** Hepatocellular carcinoma, Exosomes, Circular RNA, Nomogram, Recurrence

## Abstract

**Background:**

Liquid biopsies, for example, exosomal circular RNA (circRNA) can be used to assess potential predictive markers for hepatocellular carcinoma (HCC) in patients after curative resection. This study aimed to search for effective prognostic biomarkers for HCC in patients after surgical resection based on exosomal circRNA expression profiles. We developed two nomograms incorporating circRNAs to predict the postoperative recurrence-free survival (RFS) and overall survival (OS) of HCC patients.

**Method:**

Plasma exosomes isolated from HCC patients and healthy individuals were used for circRNA microarray analysis to explore differentially expressed circRNAs. Pearson correlation analysis was used to evaluate the correlation between circRNAs and clinicopathological features. Cox regression analysis was used to explore the correlation between circRNA and postoperative survival time as well as recurrence time. A nomogram based on circRNA and clinicopathological characteristics was established and further evaluated to predict prognosis and recurrence.

**Result:**

Among 60 significantly upregulated circRNAs and 25 downregulated circRNAs, hsa_circ_0029325 was selected to verify its power for predicting HCC outcomes. The high expression level of exosomal hsa_circ_0029325 was significantly correlated with OS (P = 0.001, HR = 2.04, 95% CI 1.41–3.32) and RFS (P = 0.009, HR = 1.62, 95% CI 1.14–2.30). Among 273 HCC patients, multivariate regression analysis showed that hsa_circ_0029325 (HR = 1.96, 95% CI 1.21–3.18), tumor size (HR = 2.11, 95% CI 1.33–3.32), clinical staging (HR = 2.31, 95% CI 1.54–3.48), and tumor thrombus (HR = 1.74, 95% CI 1.12–2.7) were independent risk factors for poor prognosis in HCC patients after radical resection. These independent predictors of prognosis were incorporated into the two nomograms. The AUCs under the 1-year, 3-year, and 5-year survival and recurrence curves of the OS and RFS nomograms were 0.755, 0.749, and 0.742 and 0.702, 0.685, and 0.642, respectively. The C-index, calibration curves, and clinical decision curves showed that the two prediction models had good predictive performance. These results were verified in the validation cohort with 90 HCC patients.

**Conclusion:**

Our study established two reliable nomograms for predicting recurrence and prognosis in HCC patients. We also show that it is feasible to screen potential predictive markers for HCC after curative resection through exosomal circRNA expression profile analysis.

**Supplementary Information:**

The online version contains supplementary material available at 10.1007/s12672-024-01060-7.

## Introduction

Hepatocellular carcinoma (HCC) is a malignancy with high mortality and recurrence rates [1]. Surgical resection is one of the most effective curative treatments for most patients with HCC. However, the long-term survival rate of these patients after hepatectomy is disheartening, and the average tumor recurrence rate is as high as 70% [2, 3]. Therefore, it is extremely important to explore purposeful biomarkers for the prognosis of HCC patients undergoing surgery to facilitate early intervention to achieve an optimal clinical outcome.

Fortunately, some clinicopathological features [4] of HCC, such as nodule number, tumor capsule, and microvascular invasion (MVI), and liquid biopsies [5] of, for example, exosomes, circulating tumor cells, and circular RNAs (circRNAs), may inform the progression of the tumor and predict the relapse and survival of HCC. Notably, in the HCC dynamic ecosystem, exosomes, identified as small extracellular vesicles (EVs) with an average size of 100 nm in diameter, disseminate various types of biological information by transferring bioactive molecules, such as proteins, lipids, RNA, and DNA, to integrate the local and distant tumor microenvironments [6, 7]. Additionally, exosome-associated RNAs, DNAs, and lipid proteins provide novel insights into the prognostic value of HCC [8]. CircRNA is a circular molecule with a high degree of stability and conservation; thus, abnormal expression of these molecules plays an essential role in the proliferation, metastasis, invasion and drug resistance of liver cancer [9–11]. Notably, several recent studies have offered a more in-depth understanding of the use of exosome-enriched circRNA expression profiles in HCC as they relate to tumorigenesis, metastasis, angiogenesis, and immunomodulation and the diagnosis and treatment of HCC [12, 13]. For example, Hu et al. explored exosomal circRNA expression profiles and confirmed that exosomal circCCAR1 facilitates the growth and metastasis of HCC, diminishing the treatment efficacy and patient life span [14]. Moreover, based on the circRNA expression profile, exosomal circUHRF1-secreting HCC cells were demonstrated to accelerate malignant processes and develop resistance to PD1 therapy, seriously damaging patient prognosis [15]. Our previous laboratory data initially explored the predictive value of circRNA in exosomes in HCC [16]. Recently, altered of extracellular vesicle circRNA expression profiles have demonstrated their predictive value in a variety of cancers, such as colorectal cancer [17], gastric cancer [18] and esophageal squamous cell cancer [19]. However, there is no relevant study on its predictive value in HCC. We speculate that exosomal circRNA expression profiling may provide a potent tool for identifying accurate prognostic markers for HCC.

To the best of our knowledge, there have been few attempts to establish prognostic models of HCC after surgery based on exosome-derived circRNA expression profiling. In this study, we constructed two nomogram models for the prognosis of HCC patients following surgery, screening from a large number of differentially expressed circRNAs obtained through analysis of circRNA expression profiles, and the two models could provide more accurate prognosis in routine clinical practice.

## Materials and methods

### Patients and specimens

A total of 319 consecutive HCC tissue samples were collected after curative resection at the First Affiliated Hospital of Chongqing Medical University for construction of prognostic models. The inclusion and exclusion criteria were as follows: (1) all patients received R0 resection, (2) absence of preoperative extrahepatic metastases, (3) no preoperative anticancer treatments, and (4) complete laboratory test data, patient records and follow-up data. In all, 273 patients with HCC met these criteria and were then included in the training cohort to screen for variables and establish the nomograms. A validation cohort consisted of 90 HCC patients were selected to validate the results. The validation cohort also met the above inclusion and exclusion criteria. We also selected 90 patients as a separate validation group for external validation. The validation cohort and training cohort is the same inclusion and exclusion criteria. The inclusion and exclusion criteria were as follows: (1) all patients received R0 resection, (2) absence of preoperative extrahepatic metastases, (3) no preoperative anticancer treatments, and (4) complete laboratory test data, patient records and follow-up data. Plasma samples were collected from 3 HCC patients and 3 healthy controls for isolation of exosomes and analysis of the circRNA gene expression profile. Fresh HCC tissues were used to isolate exosomes to validate the most significantly differentially expressed circRNAs. This study was approved by the First Affiliated Hospital of Chongqing Medical University (ID: 2023-161), and written consent was obtained from all patients.

### Isolation and identification of exosomes from plasma and HCC tissues

The plasma-derived exosomes were isolated by size exclusion chromatography (SEC) methods as described previously with minor modifications [20]. Exosomes from HCC tissue samples were separated from tissue using the protocol established previously by Vella et al. [21], with minor modifications. Particle size and concentration of exosomes were analyzed by nanoparticle tracking analysis (NTA) on the ZETAVIEW analyzer, and the size and structure of exosomes were directly observed by transmission electron microscopy (TEM). Western blot analysis was performed to detect typical exosomal biomarkers (Hsp70, TSG101, CD9 and calnexin) in exosomes derived from HCC tissue samples and plasma samples [22, 23].

### Western blotting

Western blot analysis was performed to detect typical exosomal biomarkers (Hsp70, TSG101, CD9 and calnexin) in exosomes derived from HCC tissue samples and plasma samples. For western blot analysis, the exosome supernatant was denatured in 5 × sodium dodecyl sulfonate (SDS) buffer and subjected to western blot analysis (10% SDS-polyacrylamide gel electrophoresis; 50 µg protein/lane) using rabbit polyclonal antibodies against CD63 (sc-5275, Santa Cruz, CA, USA), CD9 (60,232- I-Ig, Proteintech, Rosemont, IL), HSP90 (60,318-I-Ig, Proteintech, Rosemont, IL), Alix (sc-53540, Santa Cruz, CA, USA), TSG101 (sc-13611, Santa Cruz, CA, USA) and calnexin (10427-2-AP, Promega, Madison, WI). Each experiment was separately carried out in triplicate.

### CircRNA microarray

We carried out Arraystar Human circRNA Array V2 analysis of the 6 samples, namely, 3 HCC plasma samples and 3 healthy plasma samples. Total RNA from each sample was quantified using a NanoDrop ND-1000. Sample preparation and microarray hybridization were performed based on Arraystar’s standard protocols. Briefly, total RNA was digested with RNase R (Epicenter, Inc.) to remove linear RNAs and enrich circular RNAs. Then, the enriched circular RNAs were amplified and transcribed into fluorescent cRNA utilizing a random priming method (Arraystar Super RNA Labeling Kit; Arraystar). The labeled cRNAs were hybridized onto the Arraystar Human circRNA Array V2 (8 × 15 K, Arraystar). After washing the slides, the arrays were scanned with the Agilent Scanner G2505C.

### Reverse transcription quantitative PCR (qPCR) analysis

Total RNA was extracted from HCC tissue samples with the miRNeasy Serum/Plasma Kit (QIAGEN, Germany). According to the manufacturer’s instructions, the samples were subjected to a NanoDrop 2000 spectrophotometer (Thermo Scientific, Wilmington, DE, USA) for quantification. The qualified samples were reverse transcribed into cDNA with a PrimeScript RT Reagent Kit (TaKaRa, Japan). qRT-PCR was performed with a QuanNova SYBR Green PCR Kit (QIAGEN, Germany). Glyceraldehyde-3-phosphate dehydrogenase (GAPDH), as the internal reference gene, was used to normalize quantification of the levels of circRNAs. The 2−ΔΔCt method was performed to analyze the relative RNA expression levels. All primers, purchased from Sangon Biotech (Shanghai, China), are included in Supplementary Table 1. CircPrimer [24] was used to help design primers for circRNA and to determine the specificity of the circRNA primers. The results are expressed as the mean ± standard deviation (SD) based on three repeated independent experiments.

### Tissue microarray design and in situ hybridization

The HCC tissue microarray (TMA) containing 273 and 90 HCC tissues was built as described previously, respectively [16]. TMA was subjected to diaminobenzidine (DAB) staining and in situ hybridization. Specific oligonucleotides were used as probes labeled with digoxin (Servicebio). Horseradish peroxidase (HRP)-labeled anti-digoxin antibody was combined with digoxin-labeled nucleic acid hybrid. Then, HRP catalyzed a reaction with DAB (Servicebio) to produce a brownish-yellow product insoluble in water, thus identifying the localization and quantification of the target gene-probe hybrid. The in situ hybridization experiment was carried out according to the corresponding experimental manual.

The stained sections were then reviewed and scored as follows by a pathologist with over 15 years of experience: cells with < 20% staining were scored as negative staining (− , 1); cells with 20–49% staining were scored as (+ , 2); cells with 50–74% staining were scored as (+ + , 3); and cells with 75–100% staining were scored as (+ +  + , 4). The staining color was scored as light-yellow particle (1), brown‒yellow particle (2), and brown particle (3). The final score was defined as the staining number score multiplied by the staining color score [25].

### Statistical analysis

Statistical analyses were performed with SPSS software (26.0; SPSS, Inc., Chicago, IL) and R (version 4.3.2) for Windows. Comparisons between two groups were conducted using Student’s t tests. The association between hsa_circ_0029325 expression and the clinicopathological variables of HCC patients was statistically determined using the Pearson *χ*^2^ test or Fisher’s exact test as appropriate. The Kaplan‒Meier method was used to describe the overall survival curve and recurrence curve. Cox regression analysis and the log-rank test were used to evaluate their statistical significance. An ROC curve was constructed to evaluate the diagnostic value of the circRNAs. The nomogram, decision analysis curves, calibration curves and risk score chart were plotted with the rms package in R. A two-sided P value of < 0.05 was considered statistically significant.

## Results

### Baseline characteristics of patients

**Fig. 1:**
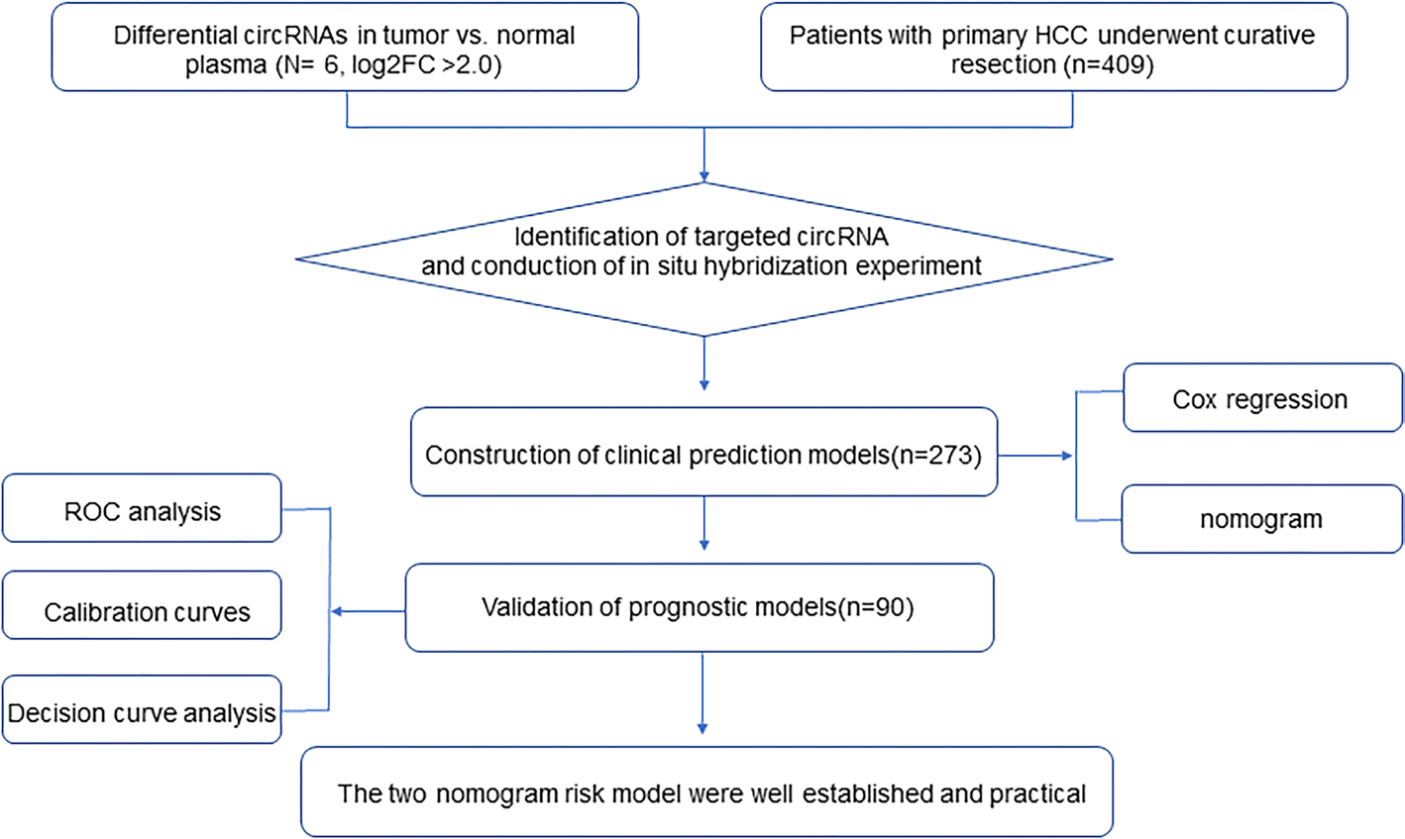
The flow chat of the study

The demographic and clinical characteristics of the patients are displayed (Table 1). There were the training cohort (n = 273) and the validation cohort (n = 90). In training cohort, the majority of patients were male (82.8%) and less than 60 years old (75.1%). Most patients had liver cirrhosis (89.1%) and hepatitis B infection (94.1%). A total of 165 (60.4%) patients had an AFP level of > 25 ng/mL, and 123 (45.1%) patients had large tumors (> 5 cm). Among these patients, more than half had no more than 2 tumors (268, 98.2%). Vascular invasion and tumor encapsulation occurred in 27.8% and 46.5% of patients, respectively. Most patients had TNM stage I disease (69.2%) and moderately differentiated tumors (72.2%). After 5 years of follow-up, the median OS and RFS were 42.4 (range, 0.43–72 months) and 35.33 months (range, 1.0–72 months), respectively. In the validation cohort, the male patients (82.22%) accounted for the majority and most patients were not over 60 years old (80.0%). Most patients had liver cirrhosis (86.67%) and hepatitis B infection (90%). In addition, 62 (68.89%) were AFP positive and 42 (46.67%) patients had large tumors (> 5 cm). Vascular invasion and tumor encapsulation occurred in 18 (20%) and72 (80%) patients in the validation cohort, respectively. 61 patients (67.78%) were in TNM stage I and 57 patients (63.33%) had moderately differentiated tumors (72.2%). It can be seen that there is no significant difference in clinicopathological features between the validation group and the test group. The flowchart of this study is shown in Fig. 1.

**Table 1:** The clinical and pathologic characteristics of 363 HCC patients with curative resection

Characteristic	Trainning (n = 273)		Validation (n = 90)		P value
Gender					
Male	226	82.80%	74	82.22%	0.43
Female	47	17.20%	16	17.78%	
Age(years)					
≤ 60	205	75.10%	72	80.00%	0.34
> 60	68	24.90%	18	20.00%	
Cirrhosis					
No	29	10.60%	12	13.33%	0.50
Yes	244	89.40%	78	86.67%	
History of hepatitis B					
No	16	5.90%	9	10.00%	0.54
Yes	257	94.10%	81	90.00%	
Total bilirubin(μmol/L)					
< 17.1	189	69.20%	66	73.33%	0.25
≥ 17.1	84	30.80%	24	26.67%	
Alanine aminotransferase(U/L)					
< 40	142	52.00%	41	45.56%	0.01
≥ 40	131	48.00%	49	54.44%	
AFP(μg/L)					
≤ 25	108	39.60%	28	31.11%	0.11
> 25	165	60.40%	62	68.89%	
Tumor size (cm)					
< 5	150	54.90%	48	53.33%	0.02
≥ 5	123	45.10%	42	46.67%	
Tumor number					
≤ 2	268	98.20%	86	95.56%	0.57
> 2	5	1.80%	4	4.44%	
Tumor thrombus					
No	197	72.20%	72	80.00%	0.30
Yes	76	27.80%	18	20.00%	
Tumor encapsulation					
No	146	53.50%	43	47.78%	0.01
Yes	127	46.50%	47	52.22%	
Tumor differentiation					
Well	5	1.80%	1	1.11%	0.36
Moderate	197	72.20%	57	63.33%	
Poor	70	25.60%	32	35.56%	
TNM stage					
I	189	69.20%	61	67.78%	0.34
II	78	28.60%	29	32.22%	
III	6	2.20%	0	0.00%	
hsa_circ_0029325					
High	160	58.60%	44	48.89%	0.06
Low	113	41.40%	46	51.11%	
Recurrence					
No	138	50.50%	37	41.11%	0.01
Yes	135	49.50%	53	58.89%	
Death					
No	188	68.90%	41	45.56%	0.22
Yes	85	31.10%	49	54.44%

### Plasma exosomal circRNA expression profile analysis

**Fig. 2:**
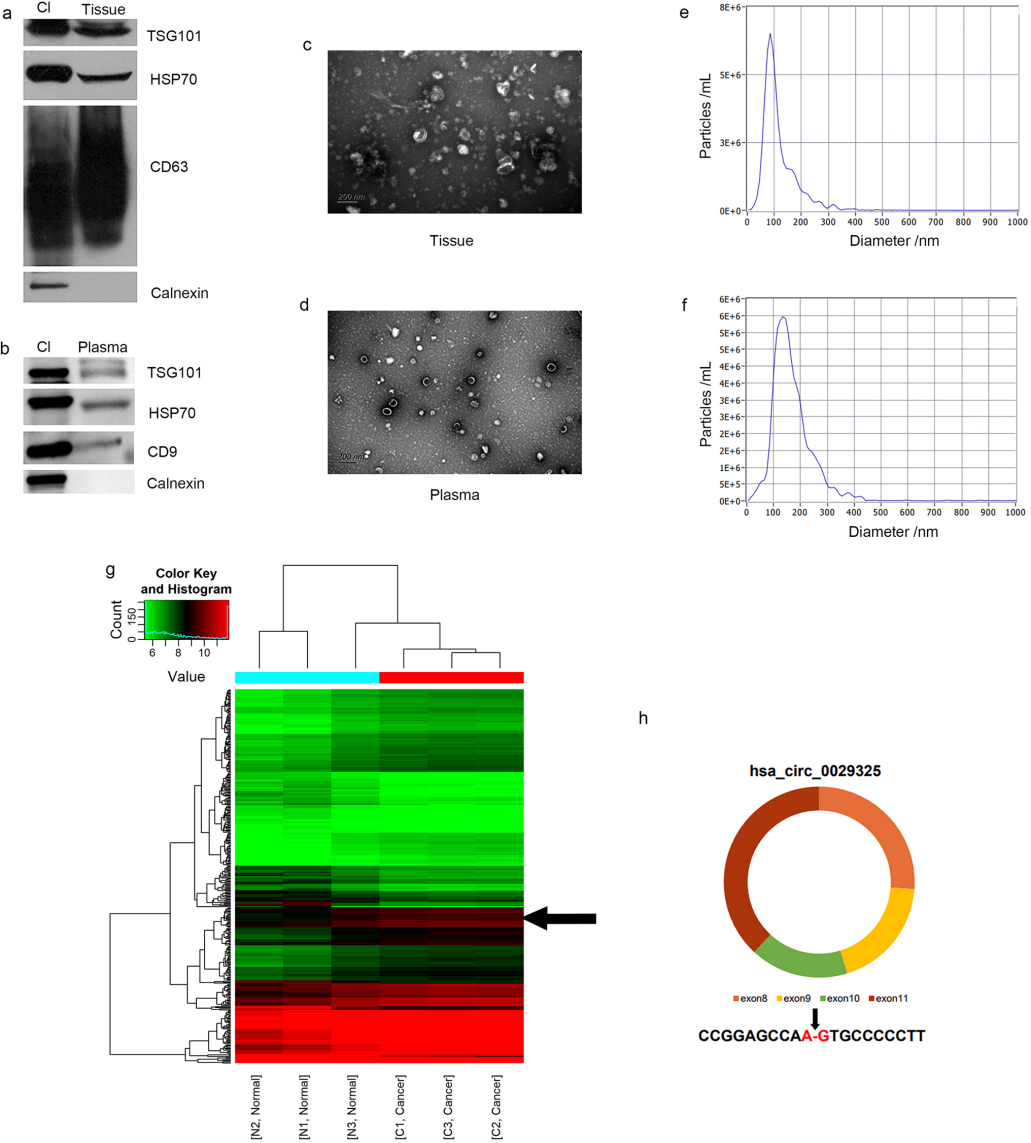
Differential exosomal circRNA in tumor vs. normal plasma. **a** and **b** A western blot for expression of typical exosomal biomarkers (Hsp70, TSG101, CD9 and calnexin). The exosomes were positive with biomarkers staining. **c** and **d** Exsomes in tissue and plasma were observed under electron microscopy. **e** and **f** The purified particles in tissue and plasma were analyzed by nanoparticle tracking analysis (NTA). **g** Heat map for the differentially expressed circRNA in the serum of hepatocellular carcinoma patients and healthy controls. **h** Schematic diagram of the structure of has_circ_0029325. Black arrow indicates the splicing junction sites

Exosomes were successfully isolated from HCC tissues and plasma and identified using TEM, a nanoparticle characterization system, and exosomal biomarkers (Fig. 2a–f). A total of 9802 plasma exosome-derived circRNAs were compared between HCC patients and healthy controls from three independent samples per group. A total of 4942 circRNAs were upregulated, and 4860 circRNAs were downregulated (Fig. 2g). Among all significantly changed circRNAs (fold change ≥ 2.0 and P < 0.05), 60 circRNAs were significantly upregulated and 25 circRNAs were downregulated in the exosomes of HCC patients compared with healthy controls. After integrating information from CIRCpedia [26] and Circular RNA Interactome [27], hsa_circ_0029325 (Fig. 2h) was selected to verify its power for predicting HCC outcomes.

### Identification and characterization of exosomes and verification of the circRNA microarray results in cancer tissue exosomes

**Fig. 3:**
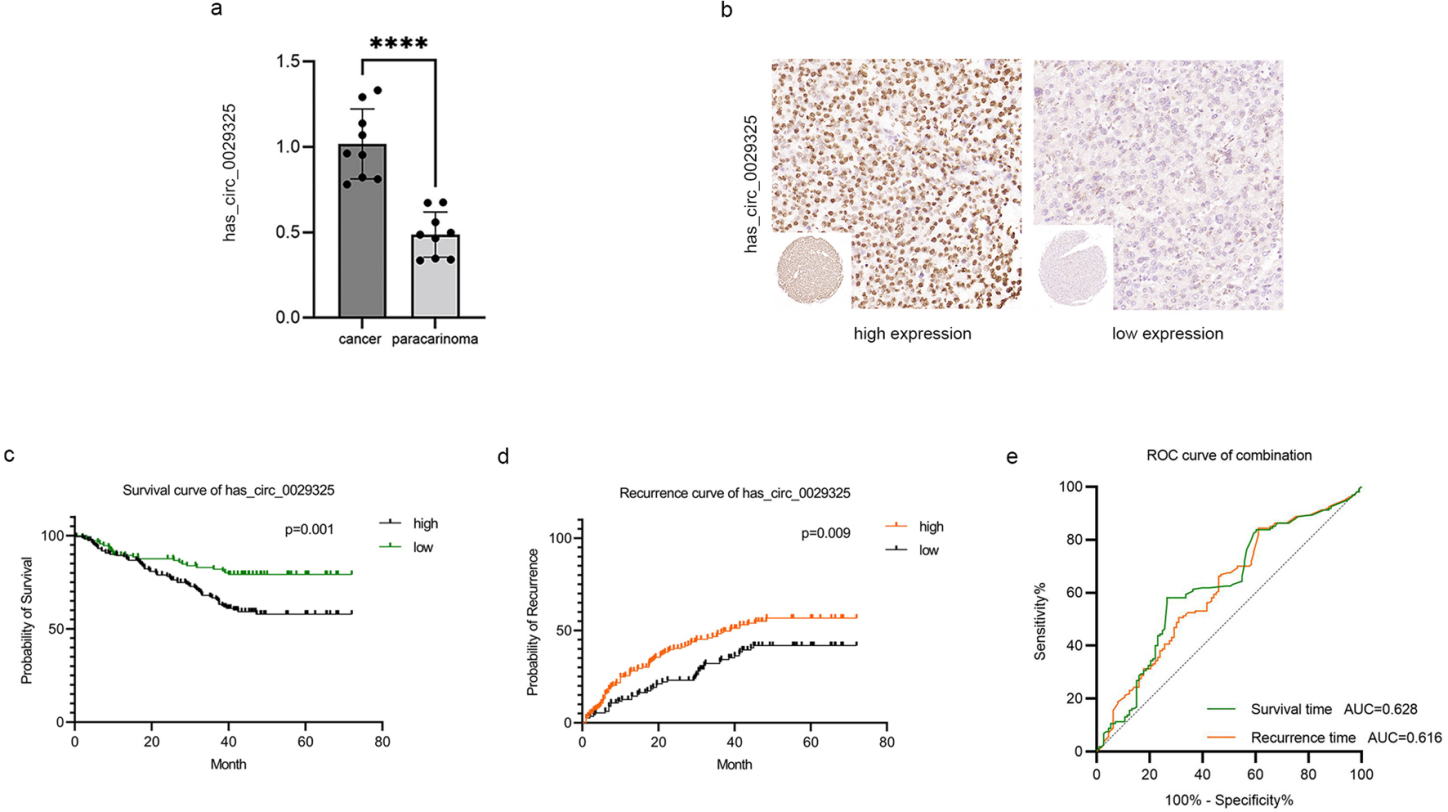
Verification of has_circ_0029325 in cancer tissue exosomes and in HCC patients. **a** The expression levels of has_circ_0029325 in HCC tissue-derived exosomes and pair paracarcinoma tissues-derived exsomes detected by qRT‐PCR method. **b** HCC tissue microarrays were used for profiling has_circ_0029325 expression by in situ hybridization. **c** and **d** Kaplan–Meier curves were used to show the overall survival and recurrence of HCC patients according to the expression of has_circ_0029325. **e** ROC curves were established to evaluate the predictive performance of has_circ_0029325 for overall survival and recurrence

After the exosomes were isolated from HCC tissues, we carried out qRT-PCR detection in these 3 pairs of tissue-secreted exosomes to quantify the expression level of circRNAs and found that hsa_circ_0029325, encapsulated within HCC tissue‑derived exosomes, was significantly upregulated compared with paired peritumoral tissue-derived exosomes (Fig. 3a).

### Correlation of hsa_circ_0029325 with clinicopathological features and prognosis

The clinical relationship between circRNA (hsa_circ_0029325) and 273 HCC patients after resection was further detected. The expression of hsa_circ_0029325 in 273 HCC tissues was examined by in situ hybridization (Fig. 3b). Based on the intensity of cell staining and the percentage of positive cells, tumor tissues were divided into the hsa_circ_0029325 high expression group (n = 160) and the hsa_circ_0029325 low expression group (n = 113, Table 2). We found that the hsa_circ_0029325 level was related only to patient recurrence (P = 0.015) and death (P = 0.001). Compared to lower hsa_circ_0029325 expression levels, higher expression levels in HCC patients were associated with lower survival and higher recurrence rates, with the ROC curve indicating that hsa_circ_0029325 had good predictive ability (Figs. 3c–e). Univariate and multivariate Cox regression was performed to screen for variables associated with recurrence time and OS, and a summary of the results is displayed in Table 3. Multivariate Cox regression analysis indicated that hsa_circ_0029325 expression was an independent predictor of postoperative recurrence time (P = 0.024, HR = 1.54, 95% CI 1.06–2.23) and overall survival (OS) (P = 0.006, HR = 1.96, 95% CI 1.21–3.18). Moreover, tumor size (P = 0.044, HR = 1.45, 95% CI 1.01–2.09; P = 0.001, HR = 2.11, 95% CI 1.33–3.32), tumor thrombus (P = 0.017, HR = 1.54, 95% CI 1.08–2.19; P = 0.0001, HR = 2.31, 95% CI 1.54–3.48), and clinical stage (P = 0.038, HR = 1.5, 95% CI 1.02–2.21; P = 0.014, HR = 1.71, 95% CI 1.12–2.70) were identified as independent risk factors for recurrence time and OS.

**Table 2:** The relationship between has-circ-0029325 expression and clinicopathologic characteristics in HCC patients

	Trainning			Validation		
Clinicopathologic variable	high(n = 160)	low(n = 113)	P value	high(n = 44)	low(n = 46)	P value
Gender						
Male	133	93	0.859	37	37	0.65
Female	27	20		7	9	
Age(years)						
≤ 60	125	80	0.168	35	37	0.916
> 60	35	33		9	9	
Cirrhosis						
No	143	101	0.999	4	8	0.247
Yes	17	12		40	38	
History of hepatitis B						
No	9	7	0.844	5	4	0.673
Yes	151	106		39	42	
Total bilirubin (μmol/L)						
< 17.1	113	76	0.553	34	32	0.408
≥ 17.1	47	37		10	14	
Alanine aminotransferase (U/L)						
< 40	85	57	0.662	18	23	0.387
≥ 40	75	56		26	23	
AFP (μg/L)						
≤ 25	58	50	0.183	13	15	0.754
> 25	102	63		31	31	
Tumor size (cm)						
< 5	81	69	0.088	18	30	0.021
≥ 5	79	44		26	16	
Tumor number						
≤ 2	158	110	0.693	41	45	0.285
> 2	2	3		3	1	
Tumor thrombus						
No	48	85	0.743	37	35	0.343
Yes	112	28		7	11	
Tumor encapsulation						
No	85	61	0.889	20	23	0.666
Yes	75	52		24	23	
Tumor differentiation						
Well	3	2		0	1	
Moderate	112	85	0.457	31	26	0.284
Poor	45	25		13	19	
TNM stage						
I	111	78		28	33	
II	48	30	0.099	16	13	0.411
III	1	5		0	0	
Recurrence						
No	71	67	0.015	15	22	0.186
Yes	89	46		29	24	
Death						
No	62	90	0.001	12	29	0.001
Yes	98	23		32	17

**Fig. 4:**
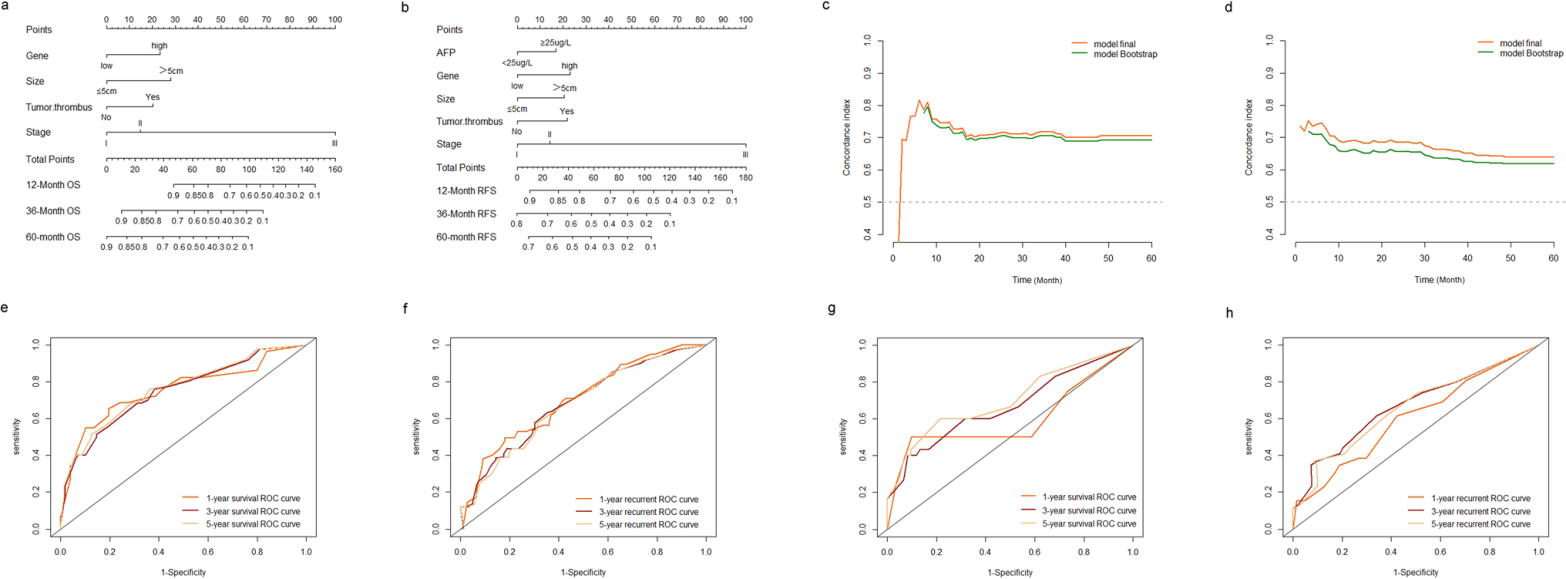
Construction and validation of the nomograms. **a** and **b** The overall survival nomogram and recurrence nomogram were established to predict the risk of HCC patients after curative resection. **c** and **d** The concordance index for the survival nomogram and recurrent nomogram indicated that the two models fits well. **e** and **f** The 1-,3-,5-year ROC curves of the survival model and recurrent model based risk features in training cohort. **g** and **h** The 1-,3-,5-year ROC curves of the survival model and recurrent model based risk features in validation cohort

**Table 3:** Univariable and multivariable cox regression the 14 differentially clinical and pathologic characteristics

Characteristics	RFS						OS					
	Univariate analysis			Multivariate analysis			Univariate analysis			Multivariate analysis		
	HR	CI95%	P value	HR	CI95%	P value	HR	CI95%	P value	HR	CI95%	P value
AFP	0.63	1.09–2.31	0.015	1.41	0.97–2.07	0.073	0.65	0.98–2.44	0.061			
Age	0.86	0.79–1.72	0.453				0.82	0.76–1.94	0.408			
Alanine aminotransferase(ALT)	0.87	0.81–1.64	0.427				0.91	0.72–1.69	0.652			
Cirrhosis	1	0.56–1.77	0.989				0.9	0.58–2.16	0.743			
Gene	0.62	1.12–2.35	0.01	1.54	1.06–2.23	0.024	0.46	1.34–3.51	0.002	1.96	1.21–3.18	0.006
History of hepatitis B	0.54	0.76–4.53	0.178				0.5	0.63–6.34	0.238			
Tumor number	1.27	0.19–3.18	0.737				0.79	0.31–5.18	0.736			
Gender	1.09	0.58–1.46	0.717				1.19	0.49–1.45	0.537			
Tumor size	0.59	1.19–2.41	0.003	1.45	1.01–2.09	0.044	0.36	1.76–4.3	0	2.11	1.33–3.32	0.001
TNM Stage	0.58	1.23–2.44	0.002	1.54	1.08–2.19	0.017	0.38	1.81–3.92	0	2.31	1.54–3.48	0
Total bilirubin(TB)	1.16	0.58–1.26	0.437				1.33	0.46–1.23	0.254			
Tumor differentiation	0.83	0.85–1.72	0.288				0.75	0.88–2.04	0.172			
Tumor encapsulation	0.78	0.9–1.82	0.162				0.82	0.79–1.86	0.369			
Tumor thrombus	0.55	1.24–2.62	0.002	1.5	1.02–2.21	0.038	0.44	1.49–3.54	0	1.74	1.12–2.7	0.014

### Development of RFS and OS nomograms and predictive performance of the nomograms

**Fig. 5:**
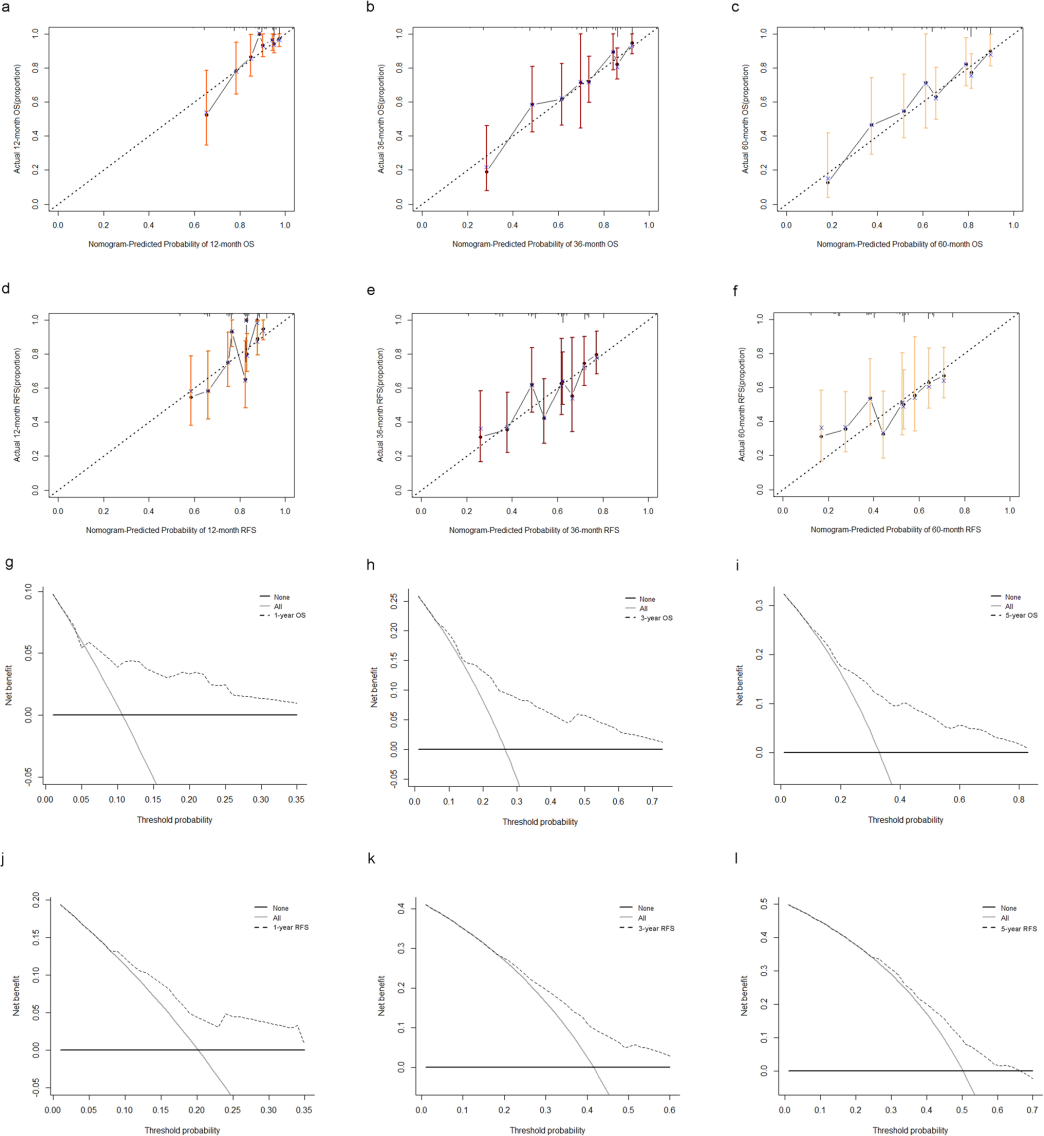
Validation of the nomograms. **A**–**c** The 1-,3-,5-year calibration curves for the overall survival nomogram in training cohort. **D**–**f** The 1-, 3-,5-year calibration curves for the recurrence nomogram in training cohort. **g**–**i** The 1-,3-,5-year clinical decision curves for the overall survival nomogram in training cohort. **J**–**l** The 1-,3-,5-year clinical decision curves for the recurrence nomogram in training cohort

The 1-, 3-, and 5-year OS nomograms (Fig. 4a) and recurrence time nomogram **(**Fig. 4b**)** were separately established, incorporating all independent hazards, including tumor size, tumor thrombus, hsa_circ_0029325, and clinical stage. The recurrence time nomogram was supplemented with an additional indicator, AFP. The bootstrap resampling method obtained a C-index that remained approximately 0.7, indicating that the two models fit well (Fig. 4c and d). The ROC curves of the two models also demonstrated their good discrimination (Fig. 4e and f). The AUC values of the 1, 3 and 5 years of the OS model in the training cohort were 0.755, 0.749, and 0.742, and those of the RFS were 0.702, 0.685 and 0.642, respectively. We also constructed a calibration curve analysis, with a perfect slope that almost coincided with the ideal curve, and the results showed the accuracy of the models (Fig. 5a–f). The clinical decision curves at 1 year, 3 years, and 5 years also indicated that a greater benefit can be obtained for patients with the use of the two models (Fig. 5g–l).

### Validation of the nomograms

**Fig. 6:**
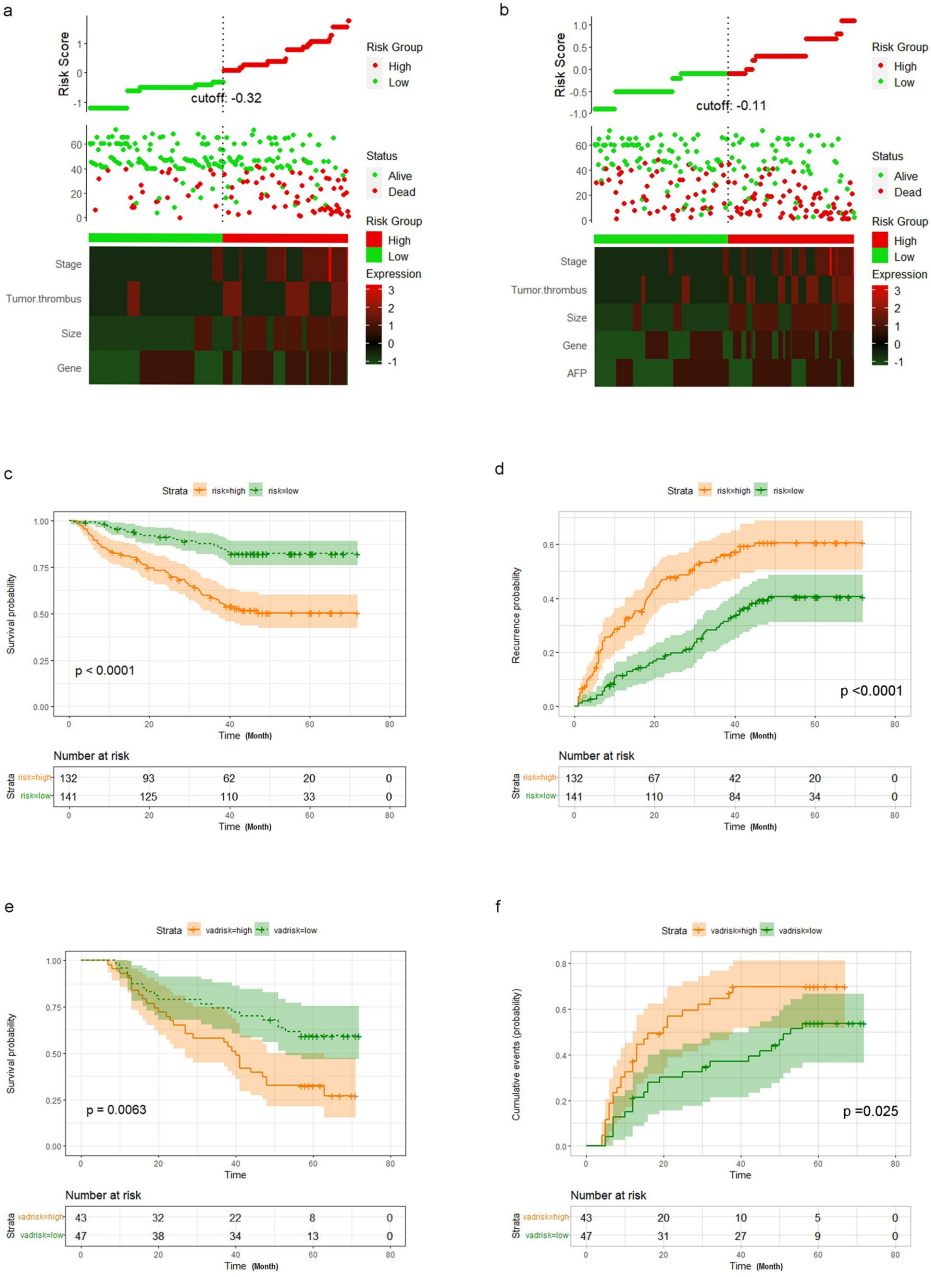
Risk score models development and validation. **a** Risk score distribution of HCC patients undergoing surgery. The heat map shows the relationship between predictive factors and overall survival(green = low risk, red = high risk). **b** Risk score distribution of HCC patients undergoing surgery. The heat map shows the relationship between predictive factors and recurrence(green = low risk, red = high risk). **c** and **d** Kaplan–Meier curves were used to show the overall survival and recurrence of high-risk(yellow) and low-risk(green) HCC patients in training cohort. **e** and **f** Kaplan–Meier curves were used to show the overall survival and recurrence of high-risk(yellow) and low-risk(green) HCC patients in validation cohort

To emphasize the reproductivity and universality of this clinical model, the bootstrap resampling method and the validation cohort are adopted for internal and external validation [28]. The AUC values of the 1, 3 and 5 years of the OS model in the validation cohort were 0.598, 0.669, and 0.690, and those of the RFS were 0.616, 0.676 and 0.632, respectively. The ROC curves (Fig. 4g and h), the calibration curve (Supplementary Fig. 1a–f) and the clinical decision curves (Supplementary Fig. 1 g–1 l) in the validation cohort (n = 90) affirm the strong discriminant ability of the nomogram. To evaluate whether postoperative patients with HCC can be effectively divided into two recommended risk groups based on this clinical model, we scored all patients and divided them into high- and low-score line groups, with significant differences in survival curves between the high- (n = 44) and low-score (n = 46) groups (Fig. 6a and b). As seen from the risk assessment table, as the risk score increased, the probabilities of recurrence and poor prognosis also increased both in the training cohort and the validation cohort (Fig. 6c–f).

## Discussion

The unique advantages of exosomes and circular RNAs have demonstrated the promise of exosomal circRNAs, as important markers for the prognosis of HCC, in the regulation and control of the malignant biological behavior of HCC [12, 29]. However, the prognostic nomogram models of HCC still require more scientific and reliable methods, especially prognostic nomograms containing genes [30, 31]. In this study, a large number of differentially expressed circRNAs were identified through the exosomal circRNA expression profile, among which hsa_circ_0029325 is highly expressed in peripheral blood and HCC tissue and is closely related to the malignant prognosis of HCC. Therefore, combined with other relevant pathological features, we established two circRNA-based nomograms for HCC, which demonstrated the precise predictive ability of prognosis and recurrence.

In the present study, we investigated the gene expression of peripheral exosome-derived circRNA in HCC patients. Many differentially regulated circRNAs were identified that may be related to the regulation of the functional properties of exosomes and trigger their activation via intercellular communication during the development of HCC. In peripheral blood, there is an efficient and strict immune monitoring system, but the liposoluble membrane structure of the exosomes can protect their internal RNAs from enzyme degradation and immune attack, for which the content of various types of RNAs in the exosomes is more stable and concentrated compared to RNAs directly exposed in peripheral blood [32, 33]. Growing evidence suggests that tumor cells produce and secrete more exosomes than normal cells and contribute to the initiation of massive malignant secretory product recruitment by various mechanisms [34–36]. Similarly, tissue exosomal circRNA can be induced to participate in the circulatory system of patients, and the resulting synergistic effects between the systemic and intratumoral microenvironments may differentially affect tumor growth.

Interestingly, our exosomal circRNA microarray found many circRNAs that were highly expressed in the peripheral blood and tissue-secreted exosomes, suggesting their potential biological behaviors in HCC. The issue of tissue specificity has been validated by two circular RNA database, [Circ Interactome (nih.gov) and CIRC pedia (yang-laboratory.com)], indicating that has_circ_0029325 is highly expressed in liver tissue, and the circRNA is closely related to the Argonaute2 (AGO2) protein. The AGO2 plays an important role in the occurrence and development of human tumors by participating in the formation of RNA induced silencing complexes, its self-catalytic and self-overexpression functions, and has gradually become a research focus [37]. We believe that has_circ_0029325 has great research potential in tumors, especially in liver cancer. Accumulating evidence has revealed that many exosome-derived circRNAs in the physiological actions of HCC contribute to tumor angiogenesis, invasion and metastasis, serving as unfavorable prognostic predictors [38, 39]. For example, exosomal circRNA-100338 promoted the metastatic ability of HCC cells by enhancing invasiveness and angiogenesis and resulted in poor survival of postsurgical HCC patients [40]. Moreover, circ-0051443, transmitted from normal cells to HCC cells through exosomes, inhibited malignant biological behavior by promoting cell apoptosis and preventing the cell cycle [41]. Herein, exosome-derived hsa_circ_0029325 reflected the modulation of tumor-promoting effects from peripheral exosomes, corresponding to the local tumor microenvironment. However, the underlying mechanisms remain to be clarified in future studies.

Here, multivariate Cox analysis showed that the expression of hsa_circ_0029325 was an independent factor predicting OS and recurrence in HCC patients, implying that differentially upregulated circRNAs derived from exosomes, such as hsa_circ_0029325, could participate in tumor progression and represent a step in the spread of protumor cellular secretory products from local to systemic distribution. Our most recent report supported this result that hepatic stellate cell (HSC)-derived exosomal circWDR25 facilitated HCC cell proliferation and invasion via the circWDR25/miR-4474-3p/ALOX15 and EMT axes [16, 42]. As a consequence, targeted therapy against exosome-derived circRNAs may be a promising therapeutic approach for this disease.

The OS and RFS nomograms might contribute to a significantly increased predictive accuracy due to incorporation of circRNA (hsa_circ_0029325) and several reliable independent clinical risk factors. In this study, although tumor size, tumor thrombus, and clinical stage could be used to stratify patients after radical surgery into different risk categories, the two nomograms showed better predictive accuracy for survival and recurrence. Finally, the C-index, calibration curve and ROC analysis also demonstrated that the OS and RFS nomograms integrating hsa_circ_0029325 filtered from the exosomal circRNA gene expression profile were accurate clinical prediction models.

We acknowledge that limitations exist in this study. First, our data came from a single medical center and comprised a small sample size. Large-sample and multicenter validation are required for prospective verification. Second, given that the HCC background is highly genetically heterogeneous, the nomograms might not be suitable for a Western population. Third, given the nature of clinical research, the in-depth tumor-promoting mechanism of these circRNAs in HCC needs to be further explored to support the rationale for the essential factor. Fourth, with the improvement of tissue exosome analysis technology, the predictive value of exosome-derived circRNAs still needs to be further validated in a larger number of patients.

## Conclusion

In summary, we demonstrated that exosomal circRNA expression profile analysis could provide a feasible approach for screening potential predictive markers for HCC in patients after curative resection. The two nomograms improved the prognosis prediction of HCC and might aid clinicians in decision-making regarding therapeutic strategies for these patients once possible signs of tumor recurrence are detected.

### Supplementary Information

Supplementary Material 1

Supplementary Material 2

Supplementary Material 3

Supplementary Material 4

## Data Availability

The data that support the findings of this study are available from the corresponding author upon reasonable request.
