# Supplementary material for: Nomograms incorporating hsa_circ_0029325 highly expressed in exosomes of hepatocellular carcinoma predict the postoperative outcomes
Source: Discov Oncol. 2024 Jun 5;15:212. doi: 10.1007/s12672-024-01060-7 (PMC11153441; doi:10.1007/s12672-024-01060-7)
Supplement: Supplementary file 1 — Supplementary Material 1 [file 12672_2024_1060_MOESM1_ESM.docx]

**
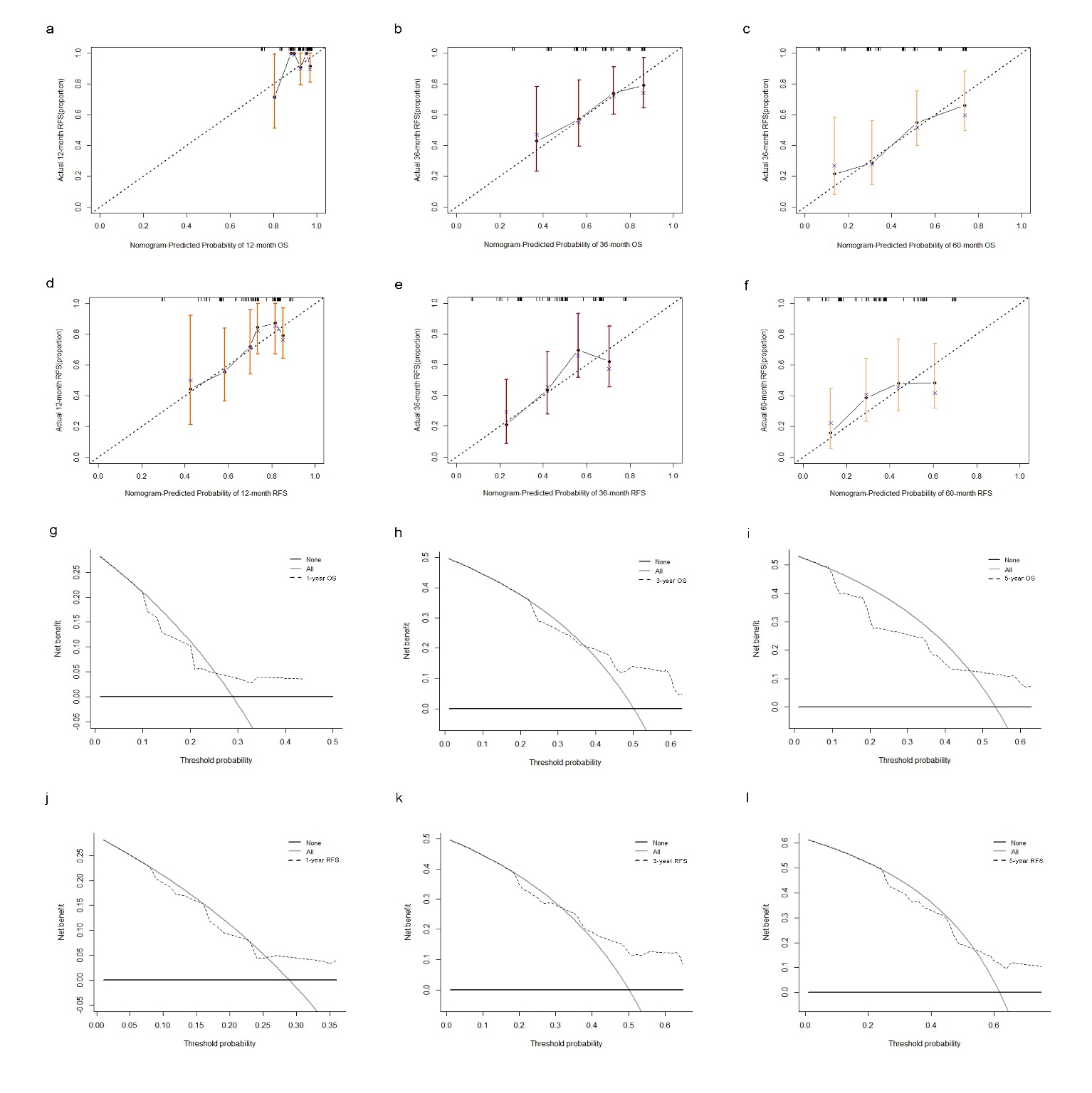
**

**Supplementary figure 1.** a-c The 1-,3-,5-year calibration curves for the overall survival nomogram in validation cohort. d-f The 1-,3-,5-year calibration curves for the recurrence nomogram in validation cohort. g-i The 1-,3-,5-year clinical decision curves for the overall survival nomogram in validation cohort. j-l The 1-,3-,5-year clinical decision curves for the recurrence nomogram in validation cohort.
