# Supplementary material for: Nomograms incorporating hsa_circ_0029325 highly expressed in exosomes of hepatocellular carcinoma predict the postoperative outcomes
Source: Discov Oncol. 2024 Jun 5;15:212. doi: 10.1007/s12672-024-01060-7 (PMC11153441; doi:10.1007/s12672-024-01060-7)
Supplement: Supplementary file 2 — Supplementary Material 2 [file 12672_2024_1060_MOESM2_ESM.pdf]

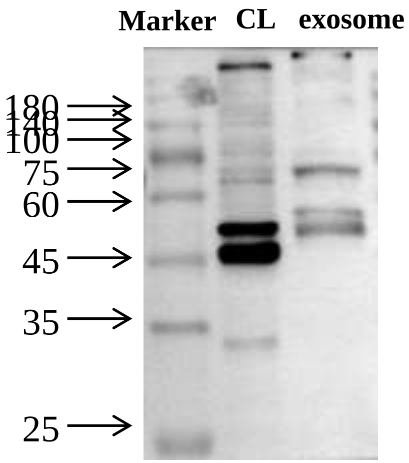

Theoretical molecular weight: 44/32 kDa,
Detected molecular weight: 45 kDa,

TSG101, Abcam, 1:1000, 45 kDa

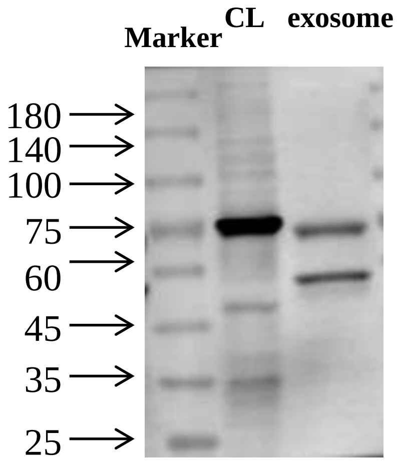

Theoretical molecular weight: 70/64 kDa,
Detected molecular weight: 70 kDa ,

HSP70, Abcam, 1:1500 , 70 kDa

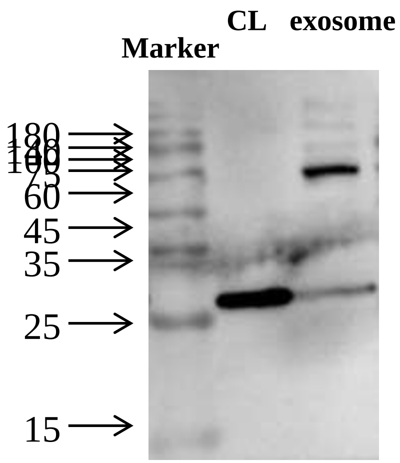

Theoretical molecular weight: 25 kDa,
Detected molecular weight: 23-27 kDa

CD9, Abcam, 1:1000, 23-27 kDa

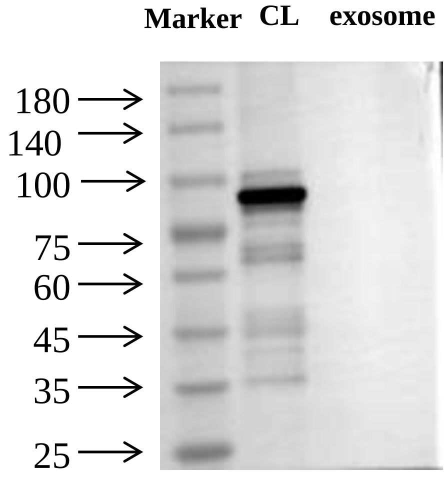

Theoretical molecular weight: 90 kDa,
Detected molecular weight: 90 kDa

Calnexin, Proteintech, 1:750, 90 kDa

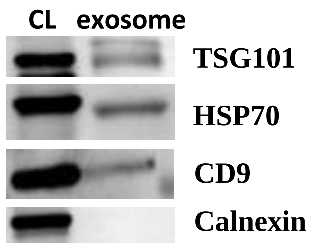
CL
exosome
TSG101
HSP70
CD9
Calnexin
