# Supplementary material for: Nomograms incorporating hsa_circ_0029325 highly expressed in exosomes of hepatocellular carcinoma predict the postoperative outcomes
Source: Discov Oncol. 2024 Jun 5;15:212. doi: 10.1007/s12672-024-01060-7 (PMC11153441; doi:10.1007/s12672-024-01060-7)
Supplement: Supplementary file 4 — Supplementary Material 4 [file 12672_2024_1060_MOESM4_ESM.pdf]

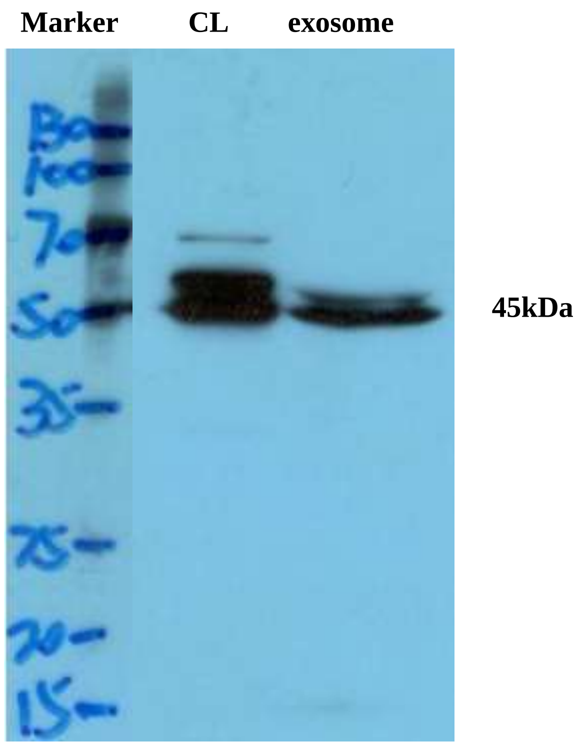

Tsg101, abcam, ab125011, 1:1000, 45KD

Marker CL exosome

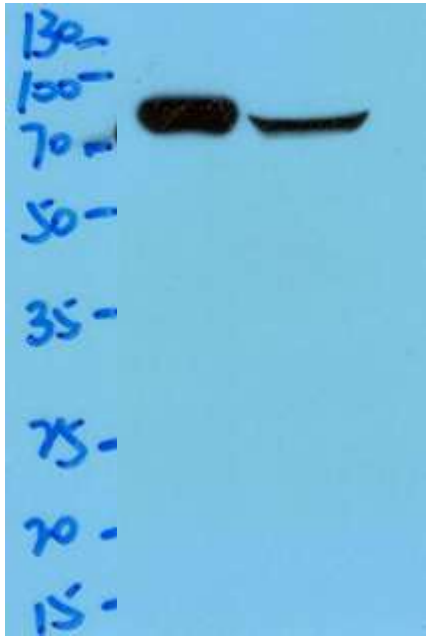

70kDa

HSP70, abcam, ab181606, 1:1000, 70KD

Marker CL exosome

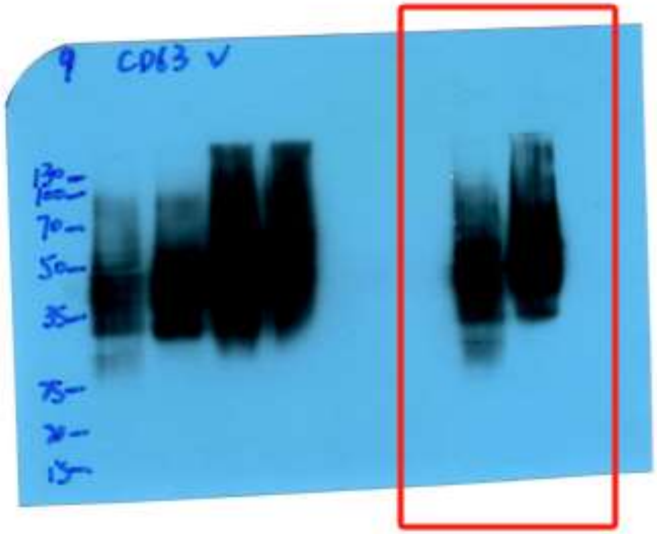

30-60kDa

CD63, Santa, sc-5275, 1:200, 30-60KD

Due to different levels of glycosylation in CD63, its bands appear dispersed

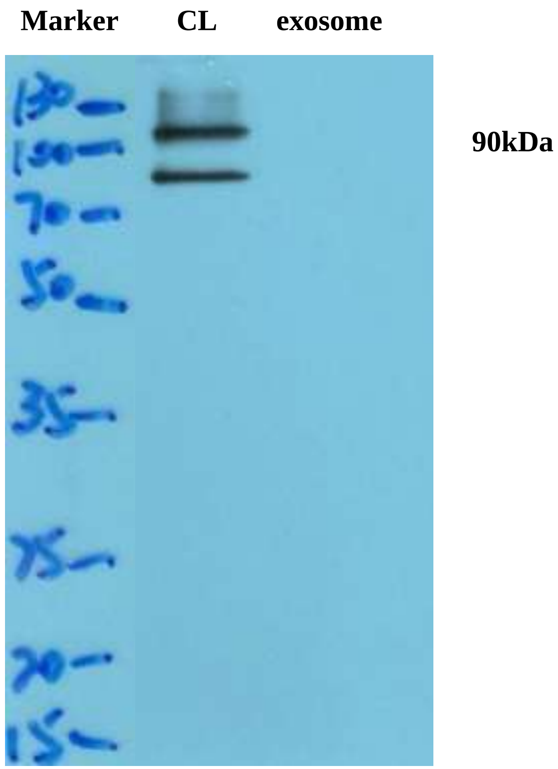

Calnexin, Proteintech, 10427-2, 1:500, 90KD

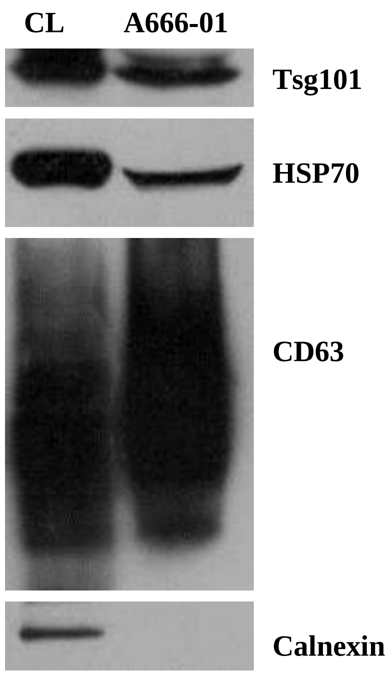
CL
A666-01
Tsg101
HSP70
CD63
Calnexin
